# Liver involvement in Gaucher disease type I: a retrospective single-center study from Ukraine

**DOI:** 10.3389/fmed.2025.1610755

**Published:** 2025-10-29

**Authors:** Nataliia Samonenko, Nataliia Olkhovych, Olena Okhotnikova, Nataliia Gorovenko

**Affiliations:** ^1^Shupyk National Healthcare University of Ukraine, Kyiv, Ukraine; ^2^National Specialized Children Hospital “Okhmatdyt”, Kyiv, Ukraine; ^3^Institute of Genetic and Regenerative Medicine, Kyiv, Ukraine

**Keywords:** liver, children, Gaucher disease, fibrosis, cirrhosis, biliary stone disease, hepatomegaly, rare diseases

## Abstract

**Background:**

Gaucher disease (GD) type I is a rare lysosomal storage disorder characterized by systemic manifestations including hepatosplenomegaly, cytopenias, and skeletal complications. Although liver involvement is common, its clinical significance and diagnostic potential remain underexplored.

**Objectives:**

To analyze the spectrum of liver involvement in Ukrainian patients with type I Gaucher disease and to evaluate its clinical, biochemical, and diagnostic significance throughout the course of the disease.

**Methods:**

We retrospectively analyzed medical records of 89 patients with confirmed GD who were monitored at the National Children’s Specialized Hospital “Okhmatdyt” (Kyiv, Ukraine) between 2001 and 2023. Type I Gaucher was confirmed in 82 patients. Diagnosis was established through enzymatic and molecular genetic testing. Liver enlargement, hepatic structural changes, laboratory markers, ultrasound findings, and serum ferritin levels were assessed, with comparative analysis between patients with and without splenectomy.

**Results:**

Hepatomegaly was observed in 91% of patients with GD type I. Despite liver enlargement, only 2.4% of patients demonstrated elevated transaminases, and no hyperbilirubinemia was reported, indicating preserved hepatic function. Additional liver pathology (steatosis, fibrosis, hemangiomatosis, cysts, or cholecystitis) was present in 45% of cases. A strong correlation was identified between the degree of hepatomegaly and elevated ferritin levels. Cholelithiasis was significantly more prevalent among splenectomized patients (42% vs. 17%; OR = 4.25; *p* = 0.014).

**Conclusion:**

Liver involvement in GD type I is common, multifaceted, and may not always be reflected in standard liver function tests. While hepatocellular function is typically preserved, structural liver abnormalities are frequently observed. Serum ferritin may serve as an auxiliary marker of disease activity. The results emphasize the need for comprehensive hepatic monitoring in GD and support the potential value of neonatal screening for early detection and management.

## Introduction

Rare inherited lysosomal storage diseases (LSDs) are a heterogeneous group of pathological conditions that includes about 70 monogenic diseases with characteristic lysosomal dysfunctions. These disorders affect the functions of numerous organs and systems, which in turn leads to a variety of progressive clinical manifestations that shorten human life. These diseases are characterized by a significant clinical polymorphism, which depends both on the degree of accumulation of uncleaved substrate in lysosomes and on the characteristics of the organs affected by the pathological process. Patients with LSD require ongoing maintenance and supportive care, and early diagnosis can significantly affect the course of the disease (1) and allow early initiation of specific therapy to prevent or delay the development of complications (2, 3).

Gaucher disease (GD) is a rare inherited metabolic disorder, namely LSD. Its incidence worldwide ranges from 1:100,000 to 1:60,000 newborns. The disease is caused by a deficiency of the lysosomal enzyme glucocerebrosidase, which leads to a progressive accumulation of glucosylceramide mainly in the liver, spleen and bone marrow, although other organs can also be affected. This is manifested by a wide range of syndromes - hepatomegaly, splenomegaly, anemia, thrombocytopenia, bone damage (3, 4).

GD is divided into three phenotypes: type 1, or non-neuronopathic; type 2, or acute neuronopathic; and type 3, or chronic neuronopathic. They differ in the presence or absence, degree and rate of progression of neurodegeneration (3, 4).

Gaucher disease type 1 is characterized exclusively by somatic manifestations, including hepatosplenomegaly, anemia, thrombocytopenia, and bone involvement with typical bone crises. The onset can occur at any age; however, this type usually follows a slow and indolent course, which complicates early diagnosis.

Gaucher disease type 2 presents with an early and rapidly progressive course with central nervous system involvement. Affected infants typically develop severe hepatosplenomegaly with neurodegenerative features within the first months of life and usually die before the age of 2 years.

Gaucher disease type 3 represents an intermediate phenotype, combining slowly progressive somatic manifestations (hepatosplenomegaly, anemia, thrombocytopenia) with neurological involvement. The most characteristic neurological features include ataxia and cognitive impairment (3, 4).

Laboratory diagnosis of GD includes the determination of the activity of the lysosomal enzyme glucocerebrosidase in leukocytes, the activity of the enzyme chitotriosidase in plasma, the accumulation of GL-1 (glucosylphingosine) and molecular genetic testing of the *GBA1* gene. Diagnosis based on the results of a bone marrow biopsy, where “Gaucher cells” are found, currently requires clarification by the methods described above due to the large number of misdiagnoses (1, 5).

The incidence of GD and LSD in general is probably underestimated due to limited awareness of these diseases among physicians.

One of the main challenges in diagnosing GD type I is the non-specific nature of its clinical manifestations and the slow, indolent course of the disease. Symptoms such as hepatomegaly, splenomegaly, thrombocytopenia, and anemia may remain unrecognized for a long time or be misinterpreted as signs of more common conditions, including chronic hepatitis, non-alcoholic fatty liver disease, hematological, or autoimmune disorders. In such cases, patients often undergo a multistep diagnostic pathway involving numerous examinations and consultations with different specialists. According to surveys of clinicians, in approximately 16%–17% of patients the diagnosis is established only seven years or more after the onset of the first symptoms (6, 7). This results in unnecessary diagnostic and therapeutic interventions and, importantly, the loss of the opportunity to initiate specific treatment at the early stages of the disease.

Liver damage is a frequent manifestation of GD and, in addition to hepatomegaly, may include steatosis, fibrosis, portal hypertension and, rarely, cirrhosis (8–10). However, direct hepatocellular damage in GD is rarely observed, as evidenced by the predominantly normal levels of transaminases in most patients (8). It is believed that the degree of hepatomegaly correlates with the severity of the disease, and ferritin levels are an indicator of inflammation rather than iron overload (11). The role of splenectomy in the development of liver tissue changes and the mechanisms of fibrosis formation, including Gaucher cell infiltration and chronic inflammation, remain a subject of discussion (12, 13).

The objective of this study was to evaluate the diagnostic and prognostic significance of hepatomegaly and splenomegaly syndrome, as well as other liver abnormalities, in patients with GD type 1 in Ukraine.

## Materials and methods of the study

A retrospective clinical study was conducted by analyzing the primary documentation of 89 patients from Ukraine who were diagnosed with GD in the period from 2001 to 2023 at the Centre for Orphan Diseases and Gene Therapy of the National Children’s Specialized Hospital (NCSH) “Okhmatdyt,” Kyiv, Ukraine.

All patients were diagnosed with GD by determining the activity of the enzymes β-glucosidase in leukocytes and chitotriosidase in blood plasma using biochemical methods and molecular genetic studies of allelic variants of the *GBA1* gene using Sanger sequencing and next-generation sequencing (NGS).

Patients with GD underwent a full comprehensive examination, including an in-depth medical history, clinical examination, as well as liver examination using ultrasound diagnostics (UD), assessment of the functional state of the liver (by serum transaminase and ferritin levels) using traditional methods of research.

For the assessment of liver status, the definitions of hepatomegaly, splenomegaly, fibrosis, and steatosis were applied. Hepatomegaly was defined as liver enlargement exceeding age-adjusted norms, in particular > 1.25 times the calculated age-related liver volume (corresponding to ∼2.5% of total body weight). Splenomegaly was defined as spleen enlargement > 1.25 times the age-adjusted normal volume. The diagnosis of hepatic steatosis and fibrosis was based on imaging methods, including ultrasound screening, computed tomography (CT), and magnetic resonance imaging (MRI), although liver biopsy remains the “gold standard” in clinical practice.

Standard methods of descriptive statistics were used for statistical analysis, including the calculation of mean values, percentages and frequencies, as well as methods of comparative analysis. To assess the relationship between categorical variables, the χ^2^-test, correlation coefficient φ, and odds ratio (OR) analysis were used. Fisher’s test and the Mann-Whitney test were used to test the statistical significance of differences between groups. Data were analyzed using Statistica 7.0 (StatSoft Inc., RRID:SCR_014213), Microsoft Excel (Microsoft Corporation, RRID:SCR_016137) and MedCalc (MedCalc Software Ltd., RRID:SCR_015044).

## Study results

**FIGURE 1:**
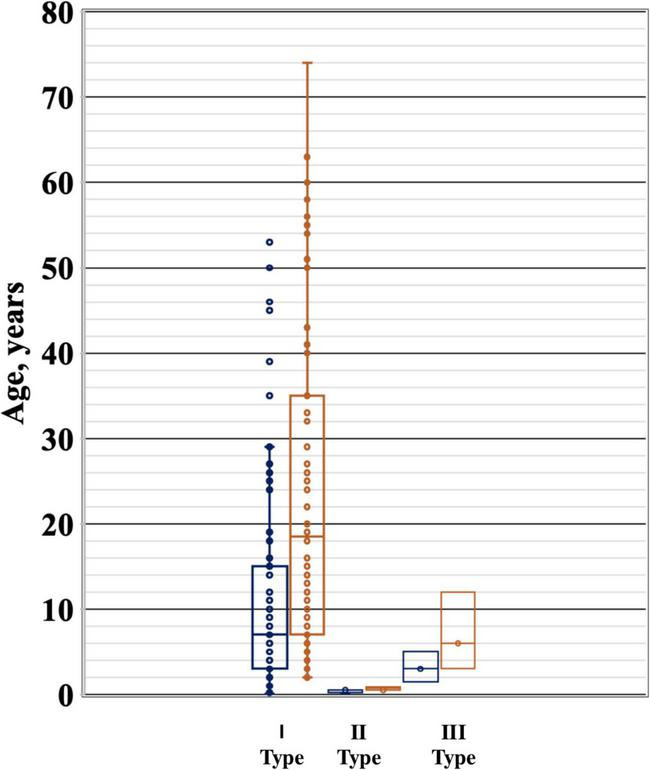
Age distribution of patients with different types of Gaucher disease.

We analyzed the medical records of 89 patients (54 females and 35 males) diagnosed with Gaucher disease (GD) at the National Children’s Specialized Hospital “Okhmatdyt” between 2002 and 2023. Among them, 82 patients (92%) were diagnosed with type I GD, four children (4.5%) with type II, and three patients (3.5%) with type III (Figure 1).

**FIGURE 2:**
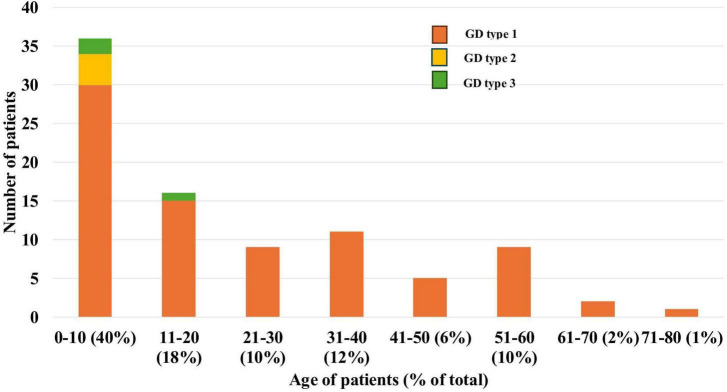
Age distribution of patients with type I Gaucher disease in Ukraine at the time of diagnosis.

The mean age at disease onset in patients with type I GD was 11.8 ± 12.53 years (range: 0.1–53 years), while the mean age at the time of diagnosis was 23.62 ± 19.01 years (range: 2–74 years) (Figure 2).

**FIGURE 3:**
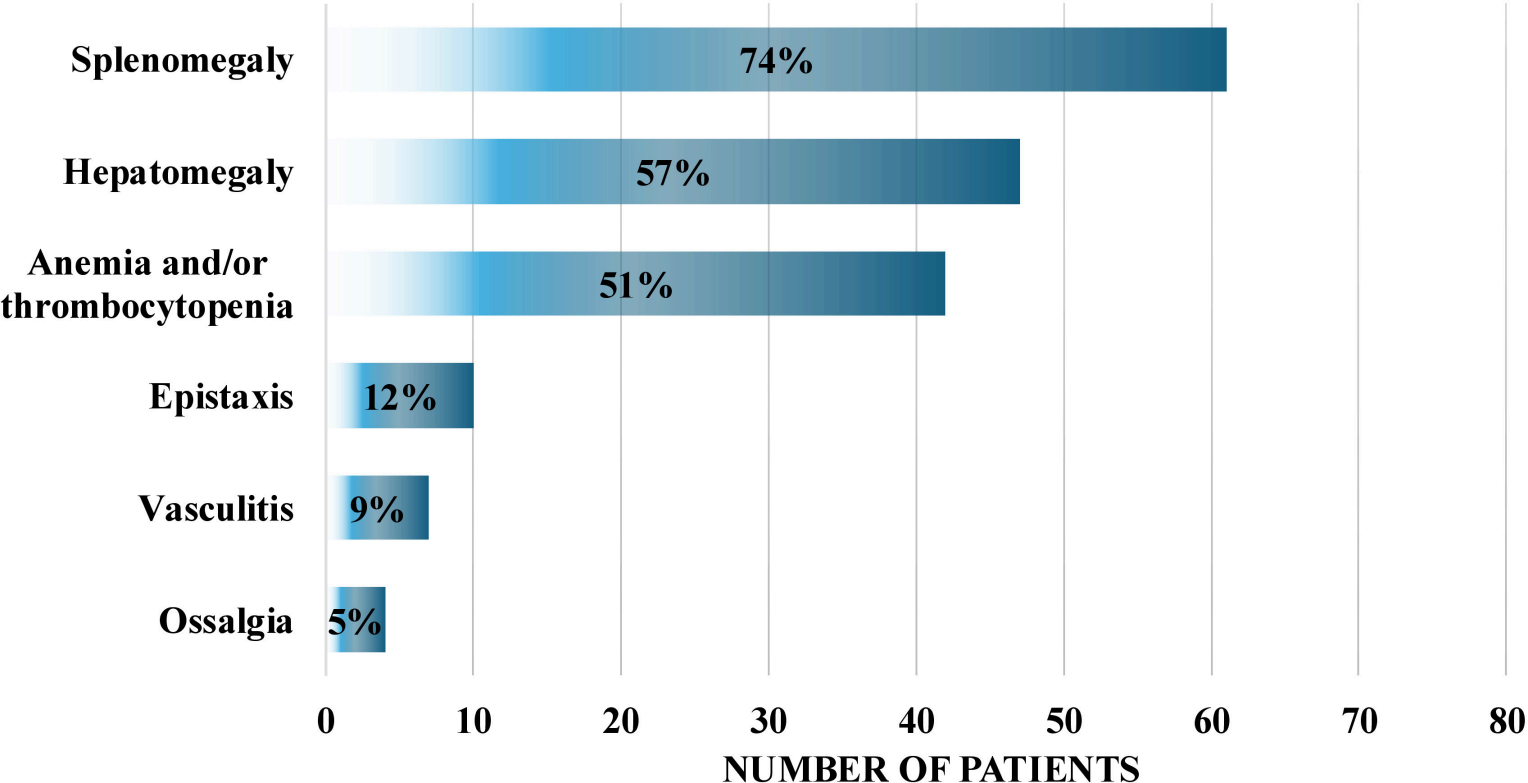
Distribution of manifestation symptoms in patients with type I Gaucher disease.

The most common initial symptoms in type I GD included splenomegaly (61 patients, 74.4%), hepatomegaly (47 patients, 57.3%), and hematological abnormalities such as anemia and thrombocytopenia (42 patients, 51.2%). Additional presenting signs included nosebleeds (10 patients, 12.2%), vasculitis-like manifestations (seven patients, 8.5%), and bone pain or ossalgia (four patients, 4.9%) (Figure 3).

**FIGURE 4:**
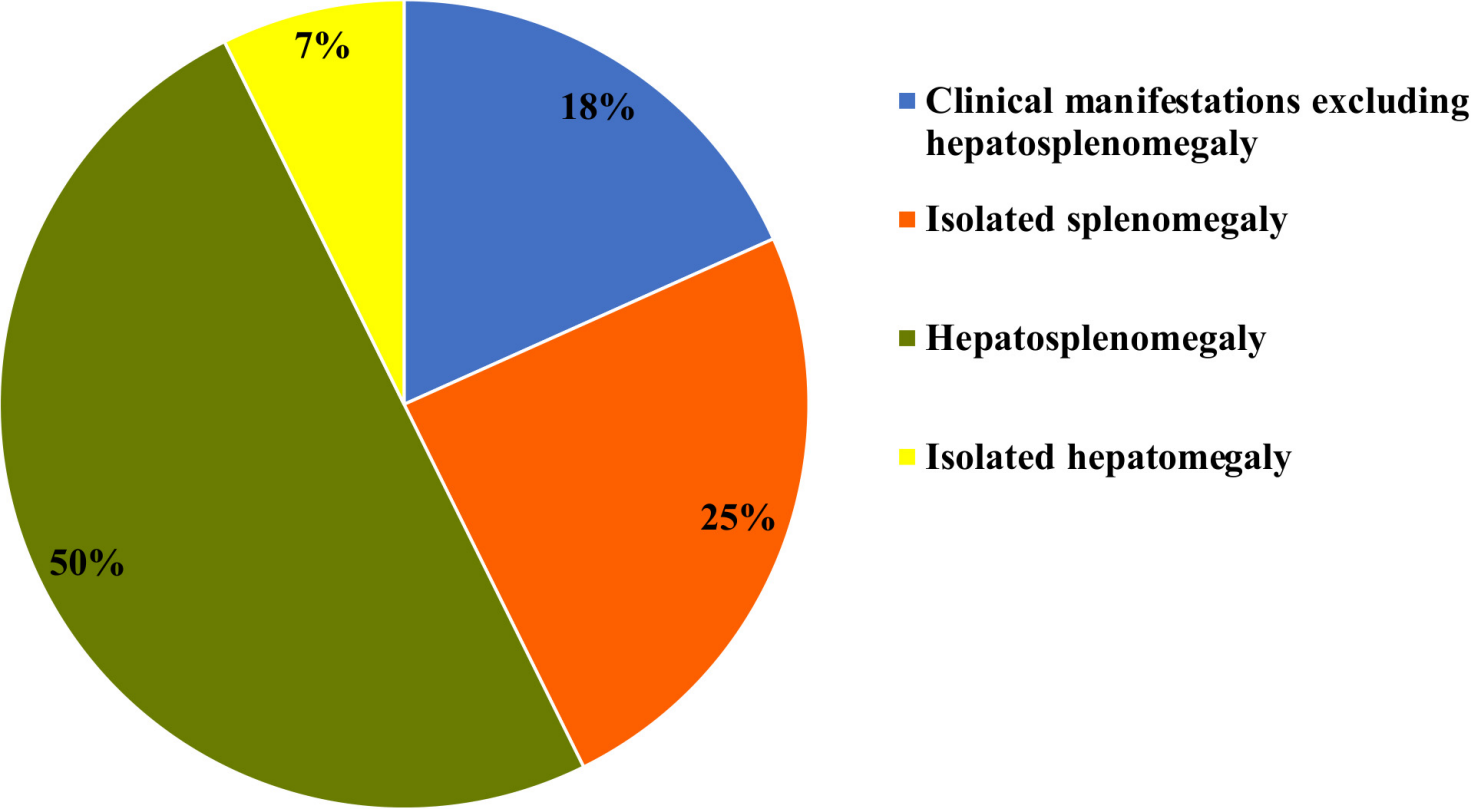
Hepatomegaly and splenomegaly patterns at initial clinical presentation of type I Gaucher disease.

At the time of clinical presentation, six patients (7.3%) had isolated hepatomegaly, 20 patients (24.4%) had isolated splenomegaly, and 41 patients (50%) presented with hepatosplenomegaly (Figure 4).

On average, 11.86 ± 14.37 years passed between the onset of the first symptoms and the establishment of a correct diagnosis in patients with type I GD. In one case, the diagnosis was made pre-symptomatically due to a positive family history (an affected sibling). In 17 patients, GD was diagnosed after the initial specialist consultation, whereas in the most delayed case, the time from symptom onset to diagnosis was 53 years.

At the time of diagnosis, 19 patients had already undergone splenectomy due to complications. The mean age at the time of spleen removal was 20.71 ± 14.03 years (range: 3.5–53 years).

Among the remaining 63 patients with type I GD, spleen volume remained within normal age-related limits in 10 patients (12.2%). In 27 patients (32.9%), spleen size exceeded normal values by more than 1.25 but less than 2.0 times, and in 26 patients (31.7%), it was more than twice the normal size.

**FIGURE 5:**
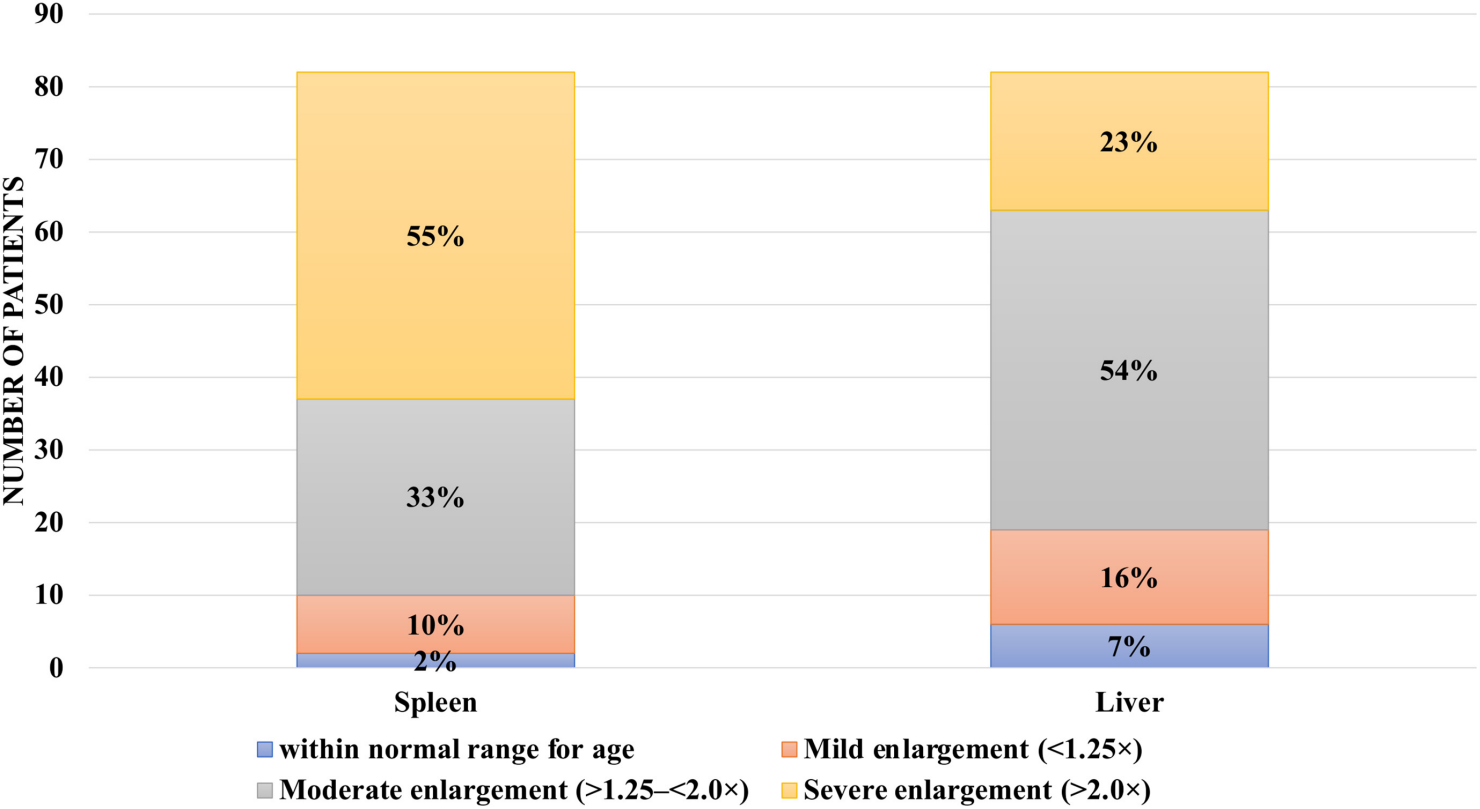
Hepatomegaly and splenomegaly among patients with type I Gaucher disease at the time of diagnosis at the National Children’s Specialized Hospital “Okhmatdyt”.

Hepatomegaly at the time of diagnosis was observed in 75 patients (91%). In seven patients (8.54%), liver size remained within normal age-related limits. In 12 patients (14.63%), liver enlargement was mild, up to 1.25 times the expected values. In 44 patients (53.66%), liver size exceeded normal values by more than 1.25 but less than 2.0 times, while in 19 patients (23.17%) liver enlargement exceeded twice the normal range (Figure 5).

**FIGURE 6:**
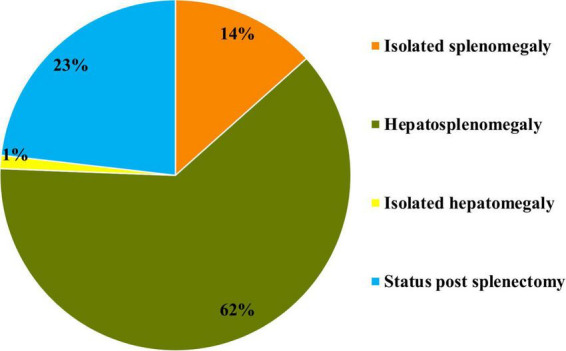
Prevalence of hepatomegaly and splenomegaly at the time of initial diagnosis in patients with type I Gaucher disease at the National Children’s Specialized Hospital “Okhmatdyt”.

At the time of diagnosis, isolated splenomegaly was observed in 11 patients (14%), isolated hepatomegaly in one patient (1%), and hepatosplenomegaly in 51 patients (62%). Additionally, 19 patients (23%) had already undergone splenectomy (Figure 6).

**FIGURE 7:**
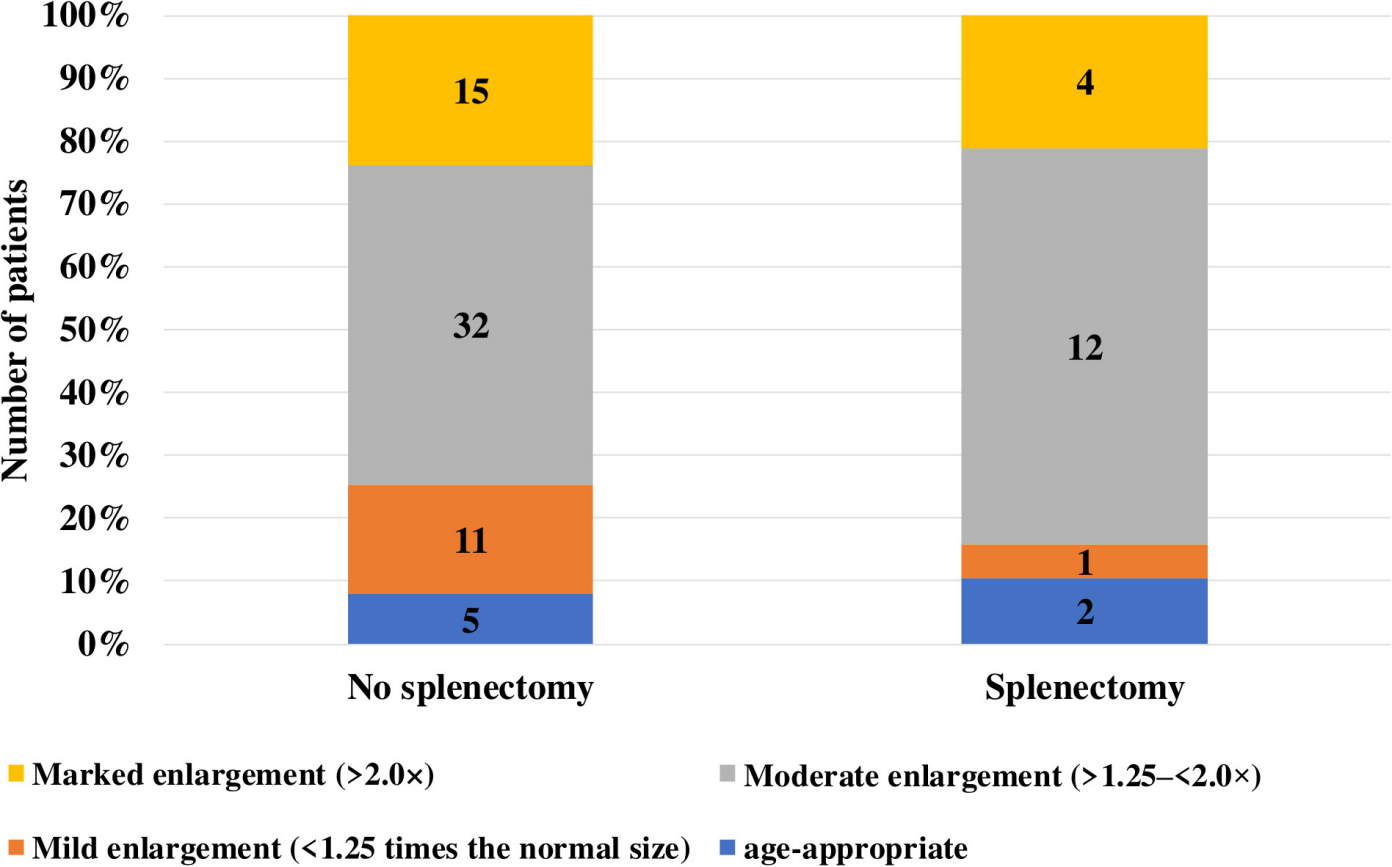
Hepatomegaly in patients with type I Gaucher disease depending on splenectomy status.

Among the 19 patients with type I GD who had undergone splenectomy, liver size remained within normal age-related limits in two patients (10%). In one patient (5%), liver enlargement was mild, up to 1.25 times the expected values. In 12 patients (63%), liver size exceeded normal by more than 1.25 but less than 2.0 times, and in four patients (21%) hepatomegaly exceeded twice the normal range (Figure 7).

Among the 63 patients who had not undergone splenectomy, liver size remained within normal age-related limits in five patients (8%). In 11 patients (17%), liver enlargement was mild (up to 1.25 times the expected values), while in 32 patients (51%) it ranged between > 1.25 and < 2.0 times. In 15 patients (24%), hepatomegaly exceeded twice the normal range.

Cholelithiasis was observed in eight splenectomized patients (42%), compared to 11 patients (17%) without splenectomy. According to Fisher’s exact test (OR = 4.25; *p* = 0.014), patients who had undergone splenectomy had a 4.25-fold higher risk of developing cholelithiasis, with the result being statistically significant, indicating a clear difference between the groups.

Other liver pathologies were observed in a limited number of patients with splenectomia: hepatic hemangioma in two patients (10%), liver fibrosis in 3 (16%), hepatic steatosis in 3 (16%), and polycystic liver disease in 3 (16%). In eight patients (42%) from this group, no liver abnormalities were identified.

At the time of diagnosis, hematological abnormalities were observed in 48 patients (58.5%) with type I GD. Among them, 21 patients (25.6%) had both thrombocytopenia and anemia, 20 patients (24.4%) had isolated thrombocytopenia, and seven patients (8.5%) had isolated anemia.

**FIGURE 8:**
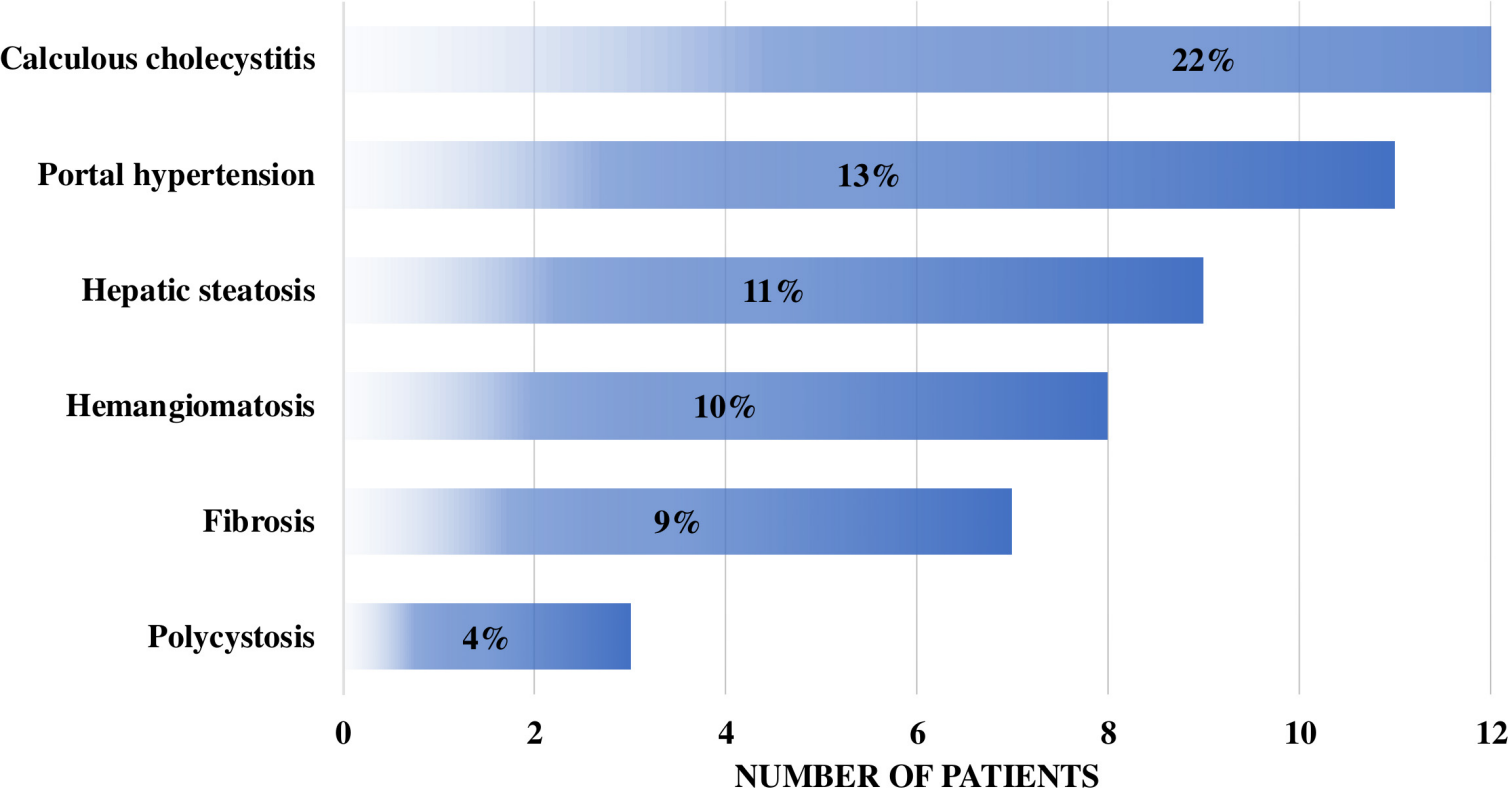
Spectrum of liver pathology in patients with type I Gaucher disease.

Various liver and hepatobiliary abnormalities were documented in 37 patients (45%) with type I GD. These included hepatic or splenic hemangiomatosis (8 out of 82 patients, 9.7%), calculous cholecystitis (18 of 82, 22%), hepatic steatosis (9 of 82, 11%), portal hypertension (11 of 82, 13.4%), liver fibrosis (7 of 82, 8.5%), and polycystic liver disease (3 of 82, 3.7%) (Figure 8).

Serum levels of transaminases and ferritin were also analyzed as potential indicators of liver involvement and functional impairment.

Among patients with type I Gaucher disease, elevated transaminase levels were observed in only 2 out of 82 patients (2.4%), and none had hyperbilirubinemia.

Elevated serum ferritin levels were found in patients with liver enlargement greater than 1.25 times normal, while no such increase was seen in those without hepatomegaly.

In patients without liver enlargement, ferritin levels remained within normal reference ranges (OR = 8.7; CI: 0.35–198.098; *p* = 0.18). In those with mild liver enlargement (< 1.25 times), no statistically significant elevation of ferritin was detected (OR = 13.46; CI: 0.5–302.16; *p* = 0.10). However, in 54% of patients with liver enlargement of 1.25–1.5 times, ferritin levels were significantly elevated (OR = 30.17; CI: 1.64–554.52; *p* = 0.002), and among those with liver enlargement > 1.5 times, 67% showed increased ferritin levels (OR = 89.28; CI: 4.16–1914.17; *p* = 0.0041) (Table 1). Due to the non-normal distribution of the measured values, the Mann–Whitney *U*-test was used to assess statistical differences between groups.

**TABLE 1:** Association between serum ferritin levels and the extent of hepatomegaly.

Patient group	Ferritin level	OR	CI (95%)	*P*-value
Without hepatomegaly	Does not exceed threshold values	8.7	0.35–198.098	0.18
With hepatomegaly < 1.25 times	No change	13.46	0.5–302.16	0.10
With hepatomegaly 1.25–1.5 times	Increased in 54% of patients	30.17	1.64–554.52	0.002
With hepatomegaly > 1.5 times	Increased in 67% of patients	89.28	4.16–1914.17	0.0041

**FIGURE 9:**
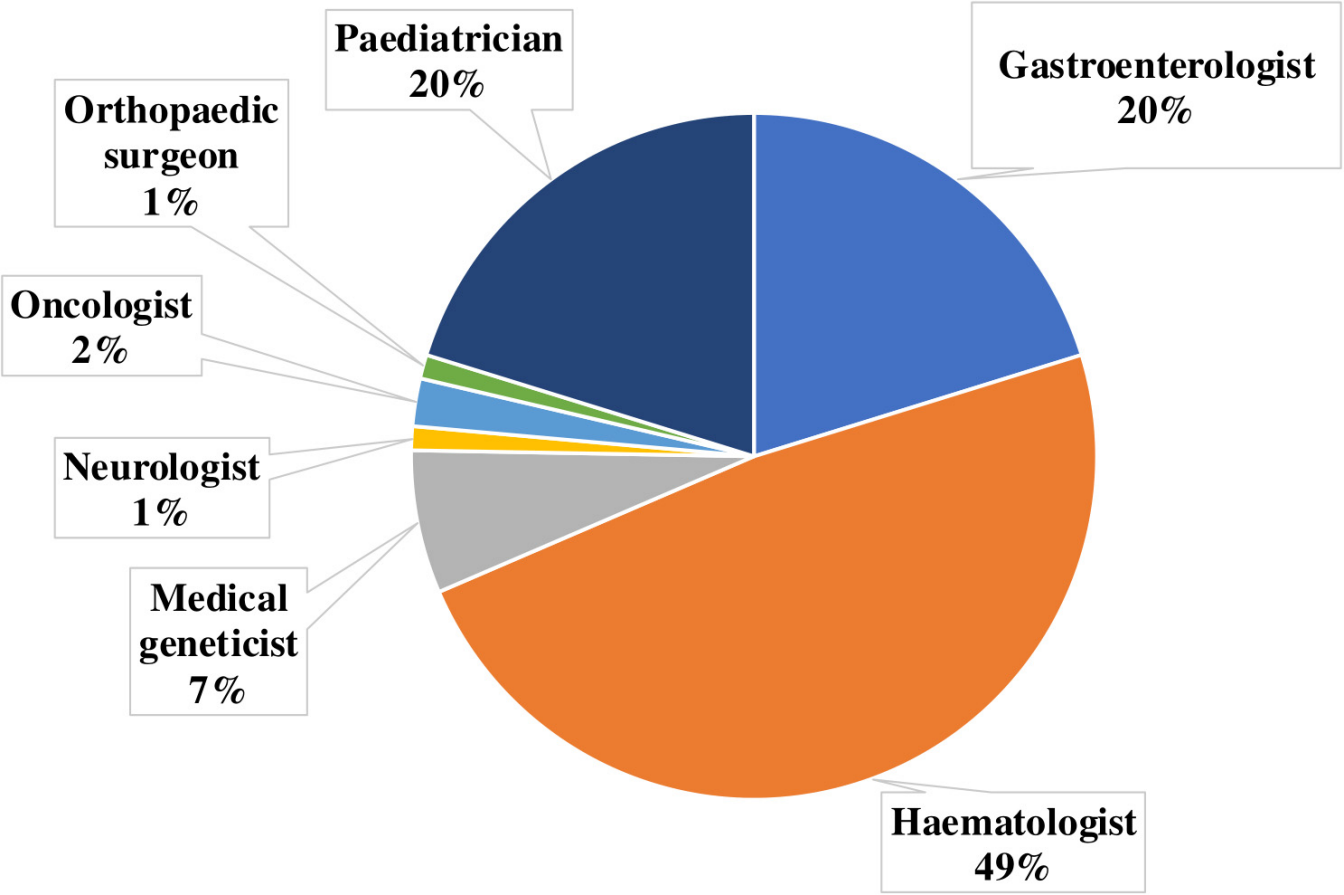
Distribution of referring physician specialties for diagnostic confirmation of Gaucher disease.

An analysis of referrals from medical specialists revealed that patients suspected of having GD were most commonly referred by hematologists (48.31%), gastroenterologists (20.22%), and pediatricians (20.22%). Referrals were also received from geneticists (6.74%), oncologists (2.25%), neurologists (1.12%), and orthopedic specialists (1.12%) (Figure 9).

All referrals from clinicians to the Center for Rare Diseases and Gene Therapy at the National Children’s Specialized Hospital “Okhmatdyt” aimed to perform comprehensive diagnostic evaluation to confirm or exclude GD. Among the referred patients, 75% had prior screening test results (β-glucosidase activity measured from dried blood spots), and 41 patients had previously undergone bone marrow aspiration in which GD was suspected based on cytological findings.

Among 82 patients, genotyping was performed in 72 (87.8%). The most common allele was c.1226A > G (p.Asn409Ser), identified in 68 of 164 alleles (39%). The second most frequent variant was c.1448T > C (p.Leu483Pro), detected in 24 of 164 alleles (14.6%). The remaining 35 variants were identified only sporadically.

## Discussion

Diagnostic testing for GD in Ukraine, including lysosomal enzyme activity measurement, was initiated in 2002 at the Laboratory of Medical Genetics of the NCSH “Okhmatdyt.” To date, this remains the only laboratory in the country capable of performing both comprehensive biochemical and molecular genetic testing for suspected LSD. Therefore, the current dataset can be considered representative and reflective of the actual distribution of GD in Ukraine, offering a valuable basis for optimizing diagnostic approaches and improving patient management strategies (14).

The distribution of GD types among Ukrainian patients generally corresponds to pan-European data from the international GD registry (9). However, in our cohort, type III GD was slightly less prevalent than type II (contrary to global statistics, where type III is more common at 5% vs. 1% for type II). This discrepancy may be due to incomplete diagnosis or specific ethnic characteristics of the population. Implementation of national screening programs would enable earlier and more accurate diagnosis of all GD types and help determine their true prevalence.

According to our analysis, the most common presenting symptoms in type I GD included splenomegaly, hepatomegaly, hematological abnormalities (anemia and thrombocytopenia), nosebleeds, and bone pain (ossalgia).

This variability in clinical presentation often leads to diagnostic errors and delays in establishing the correct diagnosis, as reflected by the wide range of time intervals observed in our analysis between symptom onset and definitive diagnosis (1).

Hepatomegaly is a common symptom among Ukrainian patients with GD and is pathogenetically associated with the accumulation of glucocerebroside in monocytes and macrophages. Additional contributing factors include inflammatory and infiltrative processes, which may disrupt hepatic tissue architecture (8). Although hepatomegaly is rarely viewed as an isolated or dominant clinical sign of GD, its diagnostic value increases when considered alongside other disease manifestations.

The results of this study did not reveal laboratory evidence of liver dysfunction, suggesting that hepatocellular integrity may be preserved despite significant liver enlargement. This supports the hypothesis that liver involvement in GD is not typically associated with direct hepatocyte damage.

In the course of this retrospective study, we characterized the clinical trajectory of type I GD, with particular focus on hepatic manifestations that may occur at different stages of disease progression.

According to international registry data, liver involvement is reported in nearly all documented cases of GD. The clinical spectrum is highly variable and may include hepatomegaly, hepatic fibrosis or cirrhosis, portal hypertension (PH), focal lesions, and cholelithiasis (9).

An increased risk of cholelithiasis in patients with GD has been documented in numerous studies (15). Gallstone disease occurs significantly more frequently in patients with GD type 1 than in the general population. Previous studies reported a prevalence of gallstones of up to 32% in a cohort of 417 patients, representing approximately a five-fold increase compared with baseline population rates (16). Identified risk factors included older age, female sex, prior splenectomy, higher body mass index, elevated LDL-cholesterol levels, and a more severe disease phenotype (17). This trend was also observed in our patient cohort. The phenomenon is likely related to changes in bile composition, particularly an increased concentration of cholesterol, which promotes the formation of cholesterol gallstones. Other proposed contributing factors include altered dietary patterns due to early satiety—leading patients to consume more calorie-dense foods—as well as chronic inflammation and prior splenectomy.

According to our data, the frequency of cholelithiasis was higher among patients who had undergone splenectomy. In our view, the influence of splenomegaly or splenectomy on the development of gallstone disease warrants further investigation.

None of the patients in our cohort exhibited hyperbilirubinemia, which may indicate preserved liver function and the absence of cholestatic syndrome or Gilbert’s syndrome.

Hepatic steatosis in GD is primarily associated with a hypermetabolic state (18). Patients with this complication require close clinical monitoring, including regular assessment of body mass index (BMI), dietary evaluation, physical activity counseling, and monitoring of blood lipid and glucose levels. According to various studies, the prevalence of steatosis in patients with type I GD ranges from 39% in Brazilian cohorts (10) to 68% in Italian patient groups (19). The relatively low prevalence of steatosis observed in our cohort may be related to ethnic and lifestyle factors, as well as to limitations in the scope and completeness of liver assessments.

Liver fibrosis, with gradual progression to cirrhosis, has also been described in patients with GD. While fibrosis is not uncommon, cirrhosis remains a relatively rare outcome. The development of fibrosis has been linked to multiple pathophysiological mechanisms, including hepatic infiltration by Gaucher cells, primary or secondary inflammatory pathways, prior splenectomy, and iron overload (8). Some studies suggest that large clusters of Gaucher cells may be fibrogenic and directly promote liver fibrosis (12, 20). Additionally, inflammation and macrophage-induced pro-inflammatory effects in GD may contribute to fibrogenesis (21, 22).

The hypothesis that splenectomy contributes to liver damage is not supported by our findings in Ukrainian patients. In our opinion, splenectomy was primarily performed in patients with advanced disease. It is more likely that hepatic involvement, regardless of severity, progresses in parallel with massive splenomegaly.

Portal hypertension (PH) may occur as a complication in severe cases of GD and can lead to life-threatening hemorrhages. However, in most cases, PH is not a consequence of liver cirrhosis but rather results from massive splenomegaly (13). Distinguishing between these two etiological mechanisms is crucial, as it directly influences therapeutic decision-making. In our Ukrainian patient cohort, PH was not observed in those who had undergone splenectomy. By contrast, all patients with PH presented with marked hepatosplenomegaly.

In clinical practice, liver involvement is commonly assessed using transaminase, bilirubin, and ferritin levels. Transaminase levels are not frequently elevated in GD, likely due to the absence of direct hepatocellular damage — a trend that was also confirmed in our study population. According to previous studies, elevated ferritin in GD primarily reflects inflammatory activity rather than iron overload, as previously believed (11), which should be considered when interpreting laboratory findings.

Although hepatomegaly and splenomegaly remain the most common hepatic manifestations of Gaucher disease, rare focal liver lesions known as Gaucheromas have also been reported in the literature. These pseudotumoral masses consist of aggregates of Gaucher cells and may mimic other hepatic pathologies on imaging, sometimes leading to diagnostic errors. Several case reports have described such findings even in pediatric patients. In our cohort of Ukrainian patients, no Gaucheromas were detected; however, awareness of these atypical manifestations remains important, particularly when evaluating focal liver lesions in the context of GD (22–25).

Patients with GD are monitored according to international and national protocols, which typically include measurements of transaminases and ferritin, as well as ultrasound (UD) evaluation of liver and spleen volumes (3, 26). However, these guidelines do not include additional assessments that may detect more subtle or advanced liver involvement. As a result, significant hepatic complications may be missed during routine follow-up.

We recommend including supplementary investigations in regular evaluations, such as screening elastography and laboratory markers (e.g., lactate dehydrogenase, bilirubin, lipid profile). While both UD and MRI are mentioned in clinical protocols for volume assessment of the liver and spleen, MRI has not shown substantial diagnostic advantage over ultrasound in our experience. Moreover, MRI is time-consuming, expensive, and has accessibility limitations (27). Therefore, we believe that ultrasound, combined with elastography, is sufficient for routine monitoring and for determining optimal management strategies in patients with GD. Nevertheless, MRI should be considered as an essential investigation at the time of diagnosis and in cases with non-specific or significant clinical manifestations.

The overall mutation spectrum observed in our cohort, with p.Asn409Ser as the most prevalent allele followed by p.Leu483Pro, is consistent with findings reported in other European populations (28, 29).

Analysis of specialist referrals for the diagnosis of GD is essential for assessing awareness of such rare conditions and optimizing the patient diagnostic pathway.

The expectedly high proportion of referrals from hematologists reflects their awareness of GD and their active use of bone marrow biopsy in differential diagnosis. Gastroenterologists and pediatricians also play an important role in referring patients, likely due to the broad clinical spectrum of GD manifestations, particularly hepatosplenomegaly.

The relatively low percentage of referrals from clinical geneticists may be explained by the fact that initial GD diagnosis is usually made at earlier stages, while geneticists in Ukraine are more often involved in coordinating care for patients with an already established diagnosis or in performing confirmatory molecular genetic testing.

The small number of referrals from neurologists and orthopedists is likely not due to a lack of clinical vigilance regarding GD, but rather to the lower prevalence of atypical forms that present with neurological or skeletal manifestations.

In general clinical practice, pediatricians, hematologists, gastroenterologists, and general practitioners often pay little attention to hepatomegaly or other liver manifestations in the context of suspected GD. The prevailing opinion is that the key signs of GD are splenomegaly accompanied by hematologic abnormalities. While interest in ossalgia as a symptom is growing, hepatic manifestations remain underrecognized, which delays early diagnosis and increases the risk of disease complications before treatment is initiated.

Currently, treatment options for type 1 GD include intravenous enzyme replacement therapy (ERT) and substrate reduction therapy (SRT). When initiated in a timely manner, such therapies lead to the elimination of nearly all major clinical manifestations of the disease, including hepatosplenomegaly, anemia, and thrombocytopenia (7). However, despite the efficacy of ERT in improving the classical clinical manifestations of GD type I, recent evidence indicates that certain hepatic complications may persist. Recent studies have shown that liver fibrosis, including mild, moderate, and even cirrhotic stages, remains prevalent both among treated and untreated patients, suggesting that ERT does not fully reverse fibrotic changes (30). Moreover, hepatic steatosis in adult patients with GD type I appears to correlate more strongly with an adverse metabolic profile than with the duration or dosage of ERT. This suggests that metabolic dysfunction associated with metabolic dysfunction–associated fatty liver disease (MAFLD) may be a key driver of long-term liver pathology, even under adequate disease-specific therapy (19, 22).

## Conclusion

Our study highlights the clinical significance of hepatic involvement in GD and its variability throughout the disease course. The heterogeneous and often indolent presentation contributes to diagnostic delays, underscoring the need for greater clinical awareness among physicians who encounter patients with hepatosplenomegaly and other non-specific signs.

Neonatal screening represents the most effective strategy for detecting the disease at a preclinical stage, enabling timely initiation of therapy and improved long-term outcomes. The development of novel biomarkers, combined with advanced imaging techniques such as elastography, offers further potential for dynamic monitoring of disease progression and personalized patient management.

In summary, improving diagnosis and care in GD relies on three key approaches: enhanced clinical awareness, neonatal screening, and integration of innovative diagnostic tools. These strategies will allow earlier identification, optimized treatment, and prevention of irreversible complications.

## Data Availability

The data analyzed in this study is subject to the following licenses/restrictions: the dataset analyzed in this study consists of fully de-identified patient data and does not contain any personally identifiable information. It is available from the corresponding author upon reasonable request. Requests to access these datasets should be directed to natalisam@gmail.com.
